# Post-transport TOPS score as a predictive marker of mortality among transported neonates and its comparative analysis with SNAP-II PE

**DOI:** 10.1016/j.heliyon.2022.e10165

**Published:** 2022-08-09

**Authors:** Shamili Pammi Ravikumar, Arivoli Kaliyan, Sathya Jeganathan, Reji Manjunathan

**Affiliations:** a Department of Paediatric, Chengalpattu Government Medical College and Hospital, Chengalpattu, Tamil Nadu, India; b Multi-disciplinary Research Unit, Chengalpattu Government Medical College, Chengalpattu, Tamil Nadu, India

**Keywords:** Neonates, Critically ill, TOPS score, SNAP II PE, Mortality

## Abstract

**Aim:**

Multiple parameters are available to predict the outcome of critically sick neonates admitted in neonatal intensive care unit (NICU). Main aim of the study is to validate the role of TOPS, especially the post-transport TOPS score as a simplified assessment of neonatal acute physiology in predicting mortality among transported neonates admitted at level III NICU. Also, to compare the efficiency of post transport TOPS score with SNAP II PE in predicting mortality.

**Methods:**

A prospective study carried out with 85 neonates transported from various primary health care centres to level III NICU. Physiological status of the neonates was assessed with the help of pre and post transport TOPS scores. Post-transport TOPS score was recorded immediately after the admission and SNAP II PE within 24 h of admission at level III NICU. Receiver operating characteristics analysis was performed to observe the mortality prediction efficiency of TOPS score and was compared with SNAP II PE.

**Results:**

64 neonates were died due to asphyxia and preterm birth (32%) related complications. Strong significant association with the mortality rate was found between the total post transport TOPS score (0.001) and SNAP II PE (0.003). The AUC, sensitivity and specificity of post transport TOPS score for a cut-off value ≤7 were 0.900, 87.5% and 80% and significant (<0.001) and for SNAP II PE for a cut-off value >12 were 0.913, 75.5% and 100% and is significant (<0.001).

**Conclusion:**

TOPS score, especially the post transport TOPS score has an equally good prediction capacity of mortality similar like SNAP II PE among mobilised critically ill neonates. Hence, the TOPS score can be used as a simple and effective method to predict mortality risk among transported neonates immediately after admission at level III NICU.

## 1 Introduction

Demand for neonatal care in India is on the rise due to increased mortality rate among the new-borns [1, 2]. In India, 70% of deliveries take place in rural areas and the transport of the preterm babies to a tertiary care centre are referred only after the birth. To overcome the adverse effects of critical illness such as respiratory distress and asphyxia among the new-borns, the babies have to be transported to a higher level neonatal intensive care unit (NICU). Unfortunately, the transport of neonates is mostly not supported by advanced monitoring facilities especially in a resource constraint environment [3, 4]. Hence, the neonatal transport system should be facilitated with advanced technological support and trained professionals who can analyse and score the changes in the physiological status of the new-borns until they reach the higher level of NICU.

Earlier, only the birth weight was considered as the most significant predictor of neonatal mortality among NICUs admission [5, 6]. Later, it was reported that the mortality rate of the neonates admitted in NICUs is also depend on the various physiological and perinatal parameters particularly related with the severity of the illness [7, 8]. Various scoring systems are available to analyse the critical illness condition of neonates within a given time frame. But, most of these scoring methods are unfortunately demand highly sophisticated equipment and this equipment are only available in high expensive large hospital settings [9, 10]. To overcome the situation, Mathur and co-workers devised a simple and rapid score system namely TOPS to assess the illness stage of tiny babies [10]. The Score for Neonatal Acute Physiology (SNAP-II) created by Richardson and colleagues is consider as one of the most common research devices used to measure the severity of illness in new-borns [8]. This score has been validated by various studies with large number of patients and has shown as a good predictor of mortality among new-borns admitted in the NICU [5, 11, 12]. The easier version of SNAP-II is named as SNAP II with Perinatal Extension (SNAP – II PE) and can standardised with the support of 6 parameters along with the added three perinatal variables such as birth weight, APGAR score and small gestational age (SGA) [11]. This score also includes not only the factors affecting the neonatal mortality in the immediate post-partum period, but also on the ante-partum and the intra-partum status of the neonates [13]. The TOPS score is a useful, simple and a reliable method that can be used in a resource limited settings during the early course of hospitalisation. The variables which are used for the scoring purpose are less susceptible to subjective variation and each variable has an independent risk associated with mortality. On the other hand, the TOPS scoring method demands large population size when compared to SNAP II and do not consider the perinatal factors [8, 14, 15].

The study is planned to delineate the physiological and demographic characteristics of the neonates transported to the Level III NICU of the central district medical college through neonatal ambulance. The study’s main objective is to evaluate the reliability of the TOPS score, such as pre (before admitting at level III NICU) and post (after admitting at level III NICU) transport TOPS as a prognostic marker to predict mortality among transported neonates in a neonatal care ambulance. We also aimed to evaluate the accuracy of the TOPS score with that of SNAP II PE in order to predict the mortality among level III NICU by identifying the differences in the physiological status of neonates while on transportation.

## 2 Method

### 2.1 Study approach

We performed a prospective analytical study among the new born population admitted at level III neonatal intensive care unit (INCU) of Department of Paediatrics, Chengalpattu Government Medical College and Hospital, Chengalpattu, Tamil Nadu, India. The study includes neonates admitted to intensive neonatal care. The neonates were transported to level III NICU through an ambulance from various community-level healthcare centres and private hospitals. The population consists of 85 neonates, and the study was conducted from June 2018 to May 2019. Neonates with congenital anomalies, home-delivered without APGAR score, and those who refused to give informed consent were excluded from the study.

**Figure 1:**
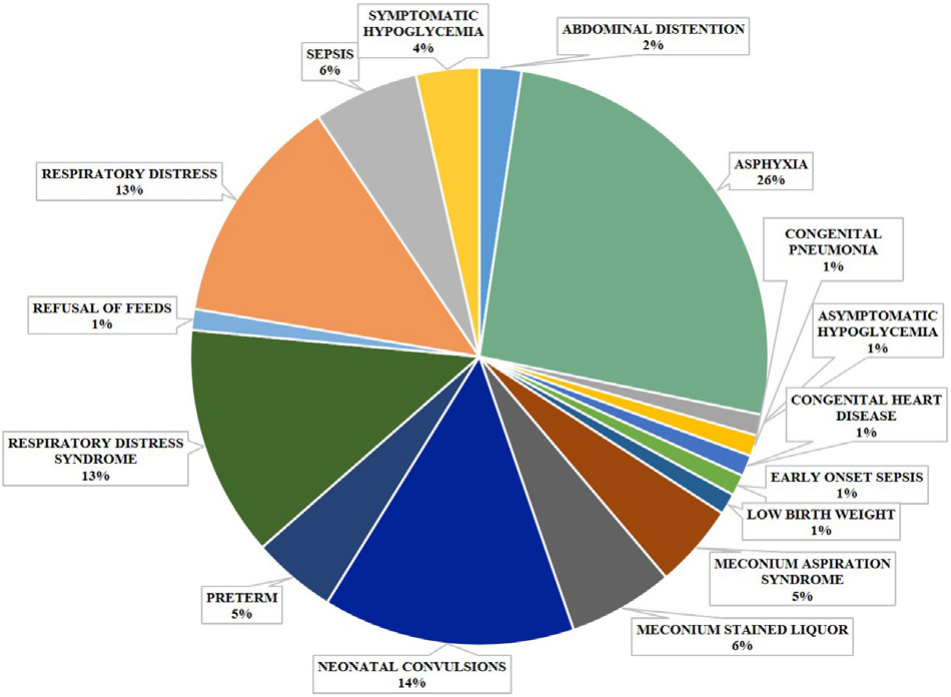
The aetiological reference of neonates included in the study.

A trained neonatal ambulance nurse assessed the physiological status of the neonates with the help of a pre-transport TOPS score. Intervention for any abnormal scoring while on transport was carried out with the support of neonatal ambulance nurses. After reaching the level III NICU, the babies were reassessed by post transport TOPS and SNAP II PE by the NICU resident. The potential of post transport TOPS score and admission SNAP II PE in predicting mortality in these neonates were assessed and compared. The changes in the physiological status of the new born during transportation were assessed from pre-and post-transport TOPS scores. The instruments used for measuring the TOPS are of standard and calibrated by the manufacturer. The instruments used for measuring TOPS score are as follows, Temperature measured by the Omron digital thermometer, Oxygen saturation by Masimo pulse oximeter, Blood sugar was evaluated by Dr Morepen Gluco one Glucometer, Perfusion done through the clinical assessment by testing over sternum/sole [11, 14]. Figure 1 represents the aetiology of referral in detail.

### 2.2 Sample size

The sample size is calculated using the Buderer’s formula as follows $\text{Sample}\;\text{size}\left(n\right)\text{based}\;\text{on}\;\text{sensitivity}=\frac{{\text{Z}}_{1-\alpha /2}^{2}\times S_{N}\times \left(1-S_{N}\right)}{L^{2}\times \mathrm{Prevalence}}$. As per the formula, the sample size is calculated 85 with 81.6% prevalence.

### 2.3 Ethical consideration

Confidentiality of the data was maintained at all times. The Institutional Ethical Committee approved the protocol (IEC) held on 27/03/2018 at the Medical Education Unit, Chengalpattu Government Medical College, and the registration number is ECR/774/INST/TN/2015.

### 2.4 Statistical analysis

Data were entered in MS Excel sheet and analyzed by SSPS software version 17. The sensitivity, specificity, positive and negative predictive value and area under the ROC curve were measured. The ROC curve depicted the predictive value of post-transport TOPS and SNAP II PE. Pre-transport and admission time TOPS scores were analyzed by Paired ‘t’ test. The Independent ‘t’ test analyzed admission TOPS score and admission SNAP II-PE at 5% level of significance. The Chi-square test was used to find the significance in categorical data.

## 3 Results

The study includes 85 neonates transported through the government neonatal ambulance service from various health care sectors to level III of NICU. The major reasons behind the reference includes, asphyxia (n = 22, 25.9%), neonatal convulsions (n = 12, 14.1%) and respiratory distress (n = 11, 12.9%). Thirty-eight among the 49 (57.6%) male candidates and 22 among the 36 (42.4%) female candidates were expired. Table 1 represents the various demographic and clinical data analysed in this study. The neonates were mainly referred from three different health care centres such as primary health care centres (n = 23, 27.1%), general hospitals (n = 57, 67.1%), and from private hospitals (n = 5, 5.9%). In association with the mortality rate, 28% of the expired neonates are referred from primary health care centres, 60% are from a government hospital, and finally the 12% of the population are from various private hospitals. We analysed the maternal complications and the mortality rate relationship. It was found that 80% of neonates admitted were born to mothers reported with no complications, and they contributed 84% of mortality. The major reasons behind the mortality are primarily pertained to preterm birth (n = 8, 32%) and asphyxia (n = 8, 32%), followed by sepsis (n = 4, 16%) and others (n = 5, 20%). Among the 85 referred neonates, 42 (49.4%) were grouped under the no resuscitation demanded category, whereas the remaining 43 (50.58%) demanded the same (Table 1). We have not found any statistically significant association between the parameters given in Table 1 with the mortality rate of transported new-borns.

**Table 1:** Demographic details.

Variables	No of population with %	survived expired	Mean ± SD	*p value*
*Gender*				
Male	49 (57.6%)	11	38	*0.185*
Female	36 (42.4%)	14	22	
*Birth weight (kgs)*				
AGA	63 (74%)	46	17	*0.406*
SGA/IUGR	19 (22%)	12	7	*0.420*
LGA	2 (4%)	1	1	*0.662*
*Gestation age (weeks)*				
Extreme preterm	1 (0.01%)	0	1	*0.529*
Very preterm	1 (12.9%)	6	5	
Moderate preterm	3 (0.3%)	2	1	
Late preterm	11 (12.9%)	7	4	
Early term	18 (21.6%)	14	4	
Term	38 (44.7%)	29	9	
Post term	3 (0.3%)	2	1	
*Mode of delivery*				
Natural labour	55 (64.7%)	37	18	
Emergency LSCS	28 (32.9%)	21	7	
Elective LSCS	2 (0.02%)	2	0	
*Resuscitation*				
Yes	43 (50.58%)			*0.459*
No	42 (49.4%)			
Initial steps	17 (20%)			
PPV	16 (18.8%)			
Chest compression	4 (4.7%)			
Intubation	5 (5.9%)			
Drugs	1 (1.2%)			
*Duration of stay*				
Survivor			4.08 ± 4.527	***P= 0.002***
Expired			15.23 ± 13.235	

The APGAR values are taken at 1st and 5th minutes after the birth were compared with the physiological outcome of neonates. The mean ± SD of APGAR taken at 1st minute (6.12 ± 5.2, p = 0.006) and 5th minutes (7.4 ± 6.44, p = 0.007) were found to be significantly associated with the outcome of the study. The mean ± SD value for the duration of the stay for survived neonates is 4.08 ± 4.527, and for expired ones is 15.23 ± 13.235 and is found to be statistically significant (p = 0.002). We try to find an association between the transport of duration and the distance travelled by the neonates with the study’s outcome. The mean ± SD values for the distance and the time of travel were given in Table 2. Almost 39% (n = 33) of the neonates reached the level III NICU within 30–60 min of referral, 31% (n = 26) were within 60–90 min, and 9% (n = 8) reached after 120 min. Among the 85 neonates, 58 (68%) neonates were transported to the hospital within 50 km of surrounding, 24 (28%) neonates were in between 50 – 100 km, and 3 neonates (0.03%) were transported from >100 km surroundings. No statistically significant association was observed between the parameters mentioned above and the outcome of the study.

**Table 2:** **Transportation characteristics**.

Variables	Population size	Mean ± SD	*p value*
*Duration of transport (Minutes)*			
Survived	60	65.75 ± 38.14	*0.774*
Expired	25	70.24 ± 35.97	
*Distance of transport (kms)*			
Survived	60	1.37 ± 38.14	*0.288*
Expired	25	1.32 ± 35.97	

SD – Standard Deviation, Chi-square Test. Pre-transport TOPS score assessed before the transportation and post-transport TOPS score assessed soon after the admission at level III NICU.

Before the babies are referred to level III NICU based on health conditions, they were stabilized either in the hospital where they were born or in the ambulance while traveling. Almost 54 (63.5%) neonates born at the government hospitals supported with pre-hospital stabilization before the admission at level III NICU. Among the remaining population (n = 18), 7 (38%) were stabilized within the ambulance with the support of a trained nurse, and all are survived. Various stages of the prior stabilization method include 1) providing 1V assessment, 2) inotrope support for managing poor circulatory conditions, and 3) respiratory support. The details of IV assessment and inotrope support were given in Table 3. We have not found any statistically significant association between the numbers of neonates who received IV assessment with the mortality rate. Depending on the degree of circulatory shock, inotrope support was provided to the neonates. It was observed that 50% (n = 4) of the mortality were recorded among those neonates who were supported with dopamine (n = 8, 9.4%) and 100% of mortality recorded among those (n = 2, 2.4%) who were supported with adrenaline. A statistically significant association (0.044) was observed between the numbers of neonates with inotropes needs and with the mortality rate. Out of the 20 neonates who were referred on inotrope support, 19 new-borns (95%) were started inotrope at the government hospital, and one neonate from the primary health care centre with the support of skilled and experienced paediatricians. In respiratory support, among the 85 neonates referred, 34 were provided with oxygen through the hood, 9 were with CPAP, and 2 were intubated. No statistically significant association was observed between the numbers of neonates with the type of respiratory access and the neonates' mortality rate. Due to the support of trained professionals, we could manage to maintain the physiological status of the neonates while on travel. Recoveries from the various issues are as follows, issues with sugar were noted among 6 neonates and were managed to maintain 100% (n = 6). SpO2 issue with 21 neonates and could manage with 8 neonates (38%), issue with perfusion noted among 14 babies and could manage with 4 neonates (28.5%), issue related with temperature noted among 32 neonates and could manage with 12 neonates (38%).

**Table 3:** Pre-transport stabilisation.

Variables	No of population with %	survived	expired	*p value*
*IV Assess*				

Yes	59 (69.4%)	43	16	*0.489*
No	26 (30.6%)	17	9	

*Inotropes*				
None	65 (76.5%)	49	16	***0.044***
Dopamine	8 (9.4%)	4	4	
Dobutamine	9 (10.6%)	7	2	
Adrenaline	2 (2.4%)	0	2	
Others	1 (1.2%)	0	1	

Both the TOPS scores (both pre and post) of the babies were accessed before and after the admission of babies at level III NICU. In the case of the pre-transport TOPS score, a statistically significant association was observed only with one variable (oxygen saturation parameter, p = 0.038). In contrast, in the case of post transport TOPS score, two variables such as oxygen saturation (0.023) and perfusion rate (0.025), were found to be significantly associated with the mortality rate of neonates under level 1 (Table 4). The SNAP II – PE of neonates, were accessed within 6 h of admission based on the physiological status of the babies. We could observe a statistically significant association with the SNAP MAP (0.097), temperature (0.001), P02/Fi02 (0.003), lowest serum pH (0.001), and the urine output (0.007) status of the babies with the outcome of the study (Table 5). The mean pre-transport TOPS score among the survived and expired babies does not show any statistically significant association with the mortality outcome. On the other hand, the post transport TOPS score (0.001) and the SNAP II-PE (0.003) show statistically significant associations with the mortality rate of neonates (Table 6).

**Table 4:** Association of TOPS score parameters with the outcome.

Variables	Pre-transport TOPS score					Post-transport TOPS score				
	Score	Population no	survived	expired	*p value*	Score	Population no	survived	expired	*p value*
Temperature	1	32	21	11	0.435	1	37	23	14	0.134
	2	53	39	14		2	48	37	11	
Oxygen saturation	1	21	11	10	**0.035**	1	23	12	11	**0.23**
	2	64	49	15		2	62	48	14	
Perfusion	1	14	9	5	0.571	1	15	7	8	**0.025**
	2	71	51	20		2	70	55	17	
Blood sugar	1	6	4	2	0.827	1	2	1	1	0.518
	2	79	56	23		2	83	59	24	

TOPS – Temperature, Oxygen saturation, Perfusion and Blood Sugar. Pre-transport TOPS score assessed before the transportation; Post-transport TOPS score assessed soon after the admission at level III NICU.

**Table 5:** SNAP II –PE parameters and its association with the outcome.

Variables	Population size	Mean ± SD	Std error	*p value*
SNAP MAP				
Expired	25	1.12 ± 4.136	0.827	***0.097***
Survived	60	0.15 ± 1.162	0.150	
Temperature				
Expired	25	4.96 ± 5.763	1.153	***0.001***
Survived	60	1.45 ± 3.382	0.437	
P02/Fi02				
Expired	25	3.84 ± 3.460	0.692	***0.003***
Survived	60	1.60 ± 2.918	0.377	
Lowest Serum pH				
Expired	25	3.60 ± 5.545	1.109	***0.001***
Survived	60	0.50 ± 2.397	0.309	
Multiple seizures				
Expired	25	5.32 ± 8.707	1.741	*0.742*
Survived	60	6.02 ± 8.913	1.151	
Urine output				
Expired	25	1.40 ± 2.291	0.458	***0.007***
Survived	60	0.33 ± 1.258	0.162	
APGAR				
Expired	25	7.20 ± 9.000	1.800	*0.466*
Survived	60	5.70 ± 8.444	1.090	
Birth weight				
Expired	25	0.40 ± 2.000	0.400	*0.122*
Survived	60	0.00 ± 0.000	0.000	
SGA				
Expired	25	3.36 ± 5.499	1.100	*0.426*
Survived	60	2.40 ± 4.841	0.625	

APGAR – “Appearance, Pulse, Grimace, Activity, and Respiration”, SGA – Small for gestational age, SD – Standard Deviation, Std – Standard, Chi-square Test.

**Table 6:** Association of Pre and Post transport TOPS scores and SNAP II PE with the outcome.

Outcome	No	Pre-transport TOPS		
		Mean ± SD	Std error	*p value*
Expired	25	6.68 ± 1.314	0.263	*0.019*
Survived	60	7.25 ± 0.836	0.108	
Expired	25	6.52 ± 1.046	0.209	*0.001*
Survived	60	7.33 ± 0.857	0.111	
Expired	25	31.20 ± 22.86	4.572	*0.003*
Survived	60	17.95 ± 15.728	2.030	

TOPS – Temperature, Oxygen saturation, Perfusion and Blood Sugar, SNAP II PE – Score for Neonatal Acute Physiology II with Perinatal Extension, SD – Standard Deviation, Std – Standard, Chi-square Test. Pre-transport TOPS score assessed before the transportation, Post-transport TOPS score assessed soon after the admission at level III NICU and SNAP II PE within 24 h of admission at level III NICU.

**Figure 2:**
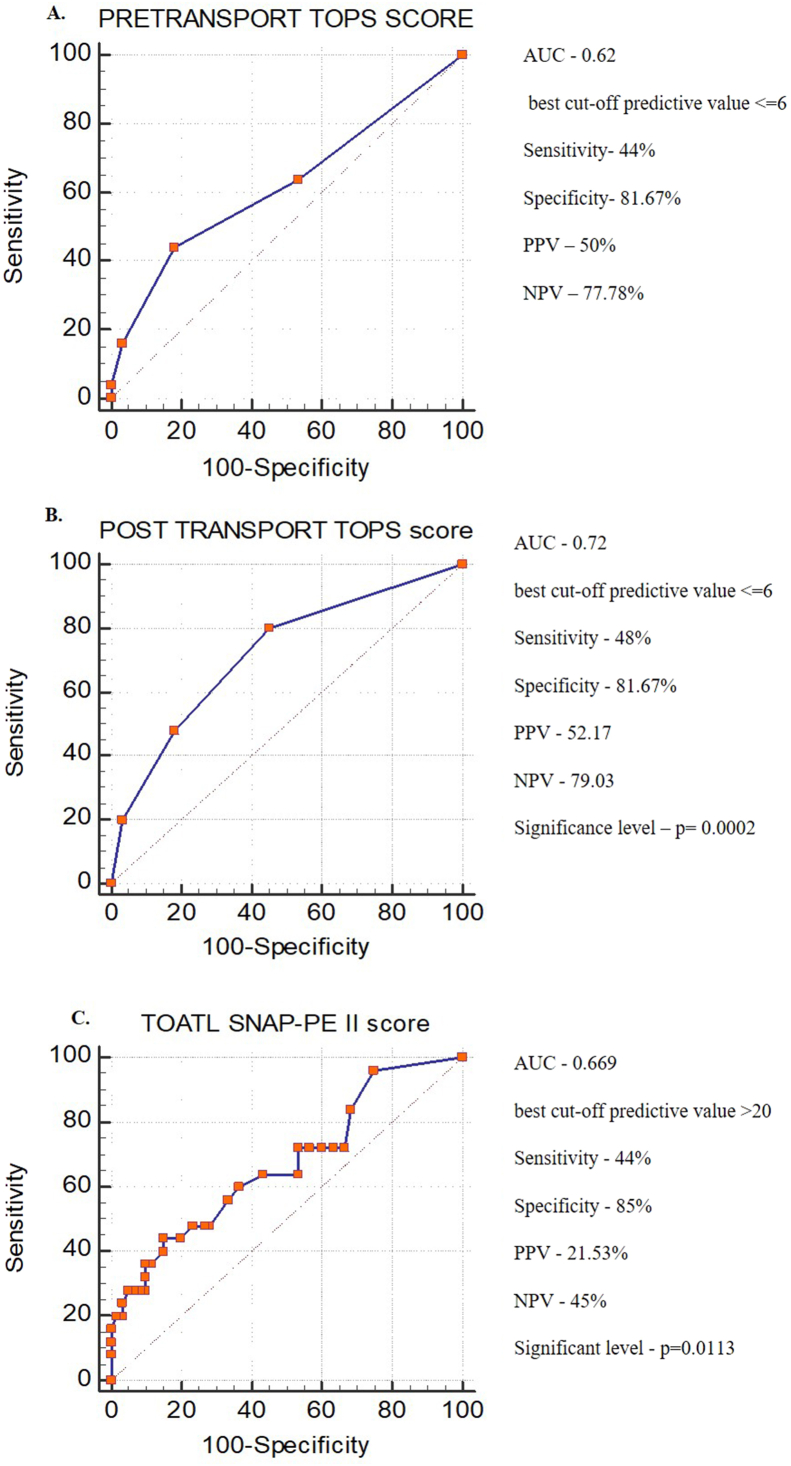
ROC curve plotted between the scores and the mortality. ROC – Receiver Operating Characteristic, AUC – Area Under the Curve, PPV – Positive Predictive Value, NPV – Negative Predictive Value, TOPS – Temperature, Oxygen saturation, Perfusion and Blood Sugar, SNAP II PE – Score for Neonatal Acute Physiology II with Perinatal Extension. Pre-transport TOPS score assessed before the transportation, Post-transport TOPS score assessed soon after the admission at level III NICU and SNAP II PE within 24 h of access.

**Figure 3:**
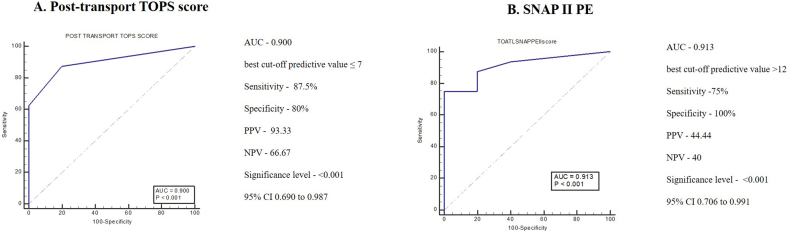
ROC curve plotted between the scores and mortality. ROC – Receiver Operating Characteristic, AUC – Area Under the Curve, PPV – Positive Predictive Value, NPV – Negative Predictive Value, TOPS – Temperature, Oxygen saturation, Perfusion and Blood Sugar, SNAP II PE – Score for Neonatal Acute Physiology II with Perinatal Extension.

Sensitivity, specificity, and accuracy scores were analysed and compared based on the Receiver Operating Characteristics (ROC). The area under the curve (AUC) in ROC for pre-transport TOPS score is 0.62 with a cut-off score 6 with 44% sensitivity and 81.67% specificity but is not significant (Figure 2A). The AUC for post-transport TOPS score is 0.72 with a cut-off score of 6 with 80% sensitivity and 55% specificity and significant (p = 0.0002) (Figure 2B). In comparison, the ROC curve for SNAP II-PE shows the AUC value of 0.669 with a cut-off score >20 with 44% sensitivity and 85% specificity with a significant value of p = 0.0113 (Figure 2C). The data indicated that the pre-transport TOPS score has poor predictive accuracy (AUC-0.62), while the post-transport TOPS score and the SNAP II-PE exhibit moderate and low predictive accuracy (AUC-0.72 and 0.669, respectively) at admission. While comparing the ability of the post transport TOPS and the SNAP II-PE in predicting the mortality within ≤72 h of admission, the first score shows an AUC of 0.900 with a cut-off score ≤7 with 87.5% of sensitivity and 80% of specificity (p < 0.001). While the SNAP II-PE shows 0.913 AUC with a cut-off score ≥12 with 75% of sensitivity and 100% of specificity (p < 0.001). The data indicates that the post-transport TOPS score is as efficient as SNAP II-PE to predict mortality in those neonates who were admitted at level III NICU after being mobilized from various health care centres through an ambulance (Figure 3).

## 4 Discussion

We found a male preponderance in this study (male – 57.6%, female – 42.4%) and is noted with less mortality rate than the female ones. This observation follows the previous reports that indicated that most of the admitted babies because of critical ills in India are male [2, 15]. Gogia *et al.* reported that there is a significant increase in institutional deliveries in India over the years [16]. Our study noted that 94% of deliveries are happened at the general hospitals and in primary health care centres. According to the previous reports, the neonatal mortality rate among mobilized new-borns is 29% and is majorly due to asphyxia and preterm birth (32%) [4, 8, 19]. In this study, we find no significant relationship between the mortality rate with the distance and duration of travel and is in oppose to previous reports that reported a direct relationship with the mortality rate and the distance travelled by the new-borns [17, 18]. This could be due to the advancement of facilities provided by the government nowadays in neonate’s ambulance care service in terms of instruments and trained professionals. All the neonates in this study are transported within an average of 100 km surroundings through facilitated neonatal ambulance services provided by the state government. Because of the promoted ambulance service, the physiological changes of the neonates were able to record and also could quickly evaluate by the professionals during the time of admission at level III NICU. This enables a rapid lifesaving process in neonates to avoid mortality and could advise the parents about the status of tiny babies.

The primary aetiology behind the referral of neonates to level III NICU in this study is associated with asphyxia (25.9%) and neonatal convulsion (14.1%) and is inconsistent with previous reports [4, 8, 19, 20]. Hypothermia is more common among neonates born with critical illness, and in our study population, 32 babies were reported with hypothermia before the transport and 37 babies after the transport. But it does not associate with the fatality of the babies and is in contrast with the previous studies [21, 22]. The babies' physiological status was supported either with IV assess, inotrope, or with respiratory supports before admission. Though we could not find any significant association between the IV assess and respiratory support with the mortality rate, a significant association was observed with the number of babies supported with inotrope such as adrenaline and dopamine with the mortality rate.

Derangements in the TOPS score have an inevitable direct role in predicting mortality among neonates reported with critical illness [20]. It was observed that under level 1 of TOPS score, change in one variable (oxygen saturation, p = 0.035) of the pre-transport TOP score and changes in two variables (oxygen saturation (0.023) and perfusion (0.25)) in post-transport TOPS are significantly correlated with the mortality rate of babies. It directly indicates that once there is an irreversible cellular injury, the efforts taken to revive the baby become ineffective.

Assessment of severity of illness is essential for health planning and the comparison of outcomes among neonates. Derangement in TOPS score highly emphasizes the fatality rate among neonates admitted at NICU. The data show a strong statistically significant association between the total post-transport TOPS score and the mortality rate among transported neonates (0.001). SNAP II PE is considered a good predictor of mortality among critically ill neonates and is directly proportional to the incident [9]. Most of the variables listed in SNAP II PE of the study are found to have a significant association with the mortality rate of transported neonates except in the case of APGAR, birth weight, and SGA variables. Among the associated variables listed in the SNAP II PE, a strong association was observed with temperature and neonates' lowest serum pH level (0.001). Thus, our analysis highly emphasizes that changes in temperature and serum pH level under SNAP II PE are good indicators for the calculation of the mortality rate in transported critically ill neonates admitted at level III NICU. The total SNAP II PE shows a strong statistically significant association with the mortality rate of transported neonates (0.003). The predictive value of both TOPS score and SNAP II PE were depicted by the ROC curve and found to have poor predictive value. Since the effect of transportation reflects the physiology of neonates within 72 h, a stratified analysis within 72 h is more acceptable. Doing so, we could find that both the TOPS score (AUC-0.900, <0.001) and SNAP II PE (AUC-0.913, <0.001) reflect a better predictive value. The post transport TOPS score is highly reliable to predict mortality among transported neonates and is almost equal to the mortality prediction efficiency of SNAP II PE.

## 5 Conclusion

TOPS score, especially the post-transport TOPS score, is a reliable test to predict mortality among transported neonates within 72 h of admission if the environment is not supported enough with the facilities. The evaluation is as efficient as SNAP II PE, which can be done only with infra-structures available at NICU. TOPS score can be assessed easily at the bedside immediately after admission. Thus, the post TOPS score can be used effectively to sort babies based on the required prompt treatment and could aim for the intact survival of critically ill and mobilized neonates. Since the neonates' physiological status is badly affected by the transport, it is highly recommended to have the baby’s TOPS score assessment at the referral centres, before transport, and on arrival at NICU with the aid of skilled health care professionals for the best results.

## Declarations

### Author contribution statement

Shamili Pammi Ravikumar: Conceived and designed the experiments; Performed the experiments.

Arivoli Kaliyan: Conceived and designed the experiments.

Sathya Jeganathan: Contributed reagents, materials, analysis tools or data.

Reji Manjunathan: Analyzed and interpreted the data; Wrote the paper.

### Funding statement

This research did not receive any specific grant from funding agencies in the public, commercial, or not-for-profit sectors.

### Data availability statement

Data included in article/supp. material/referenced in article.

### Declaration of interest’s statement

The authors declare no conflict of interest.

### Additional information

No additional information is available for this paper.
